# Supplementary material for: Protective Effect of Astaxanthin on Ochratoxin A-Induced Kidney Injury to Mice by Regulating Oxidative Stress-Related NRF2/KEAP1 Pathway
Source: Molecules. 2020 Mar 18;25(6):1386. doi: 10.3390/molecules25061386 (PMC7144393; doi:10.3390/molecules25061386)
Supplement: Supplementary file 1 [file molecules-25-01386-s001.pdf]

Protective Effect of Astaxanthin on Ochratoxin A-Induced Kidney Injury to Mice by Regulating Oxidative Stress-Related NRF2/KEAP1 Pathway

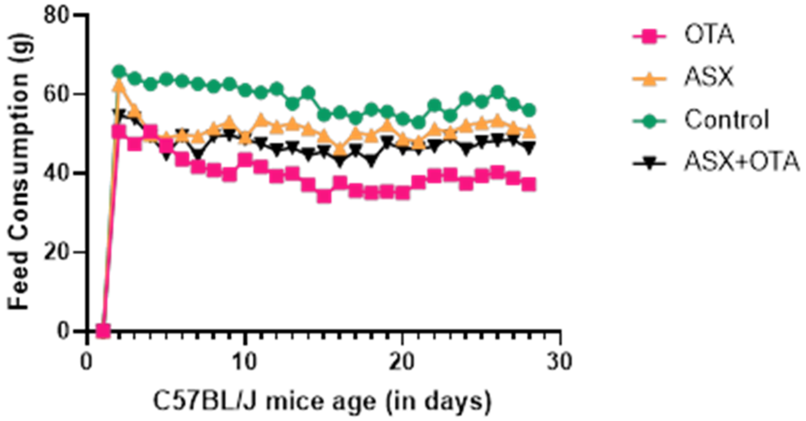

Figure S1. Feed conversion of C57BL/J mice from day 1 to day 27. n=20 mice/group.
